# Photon versus carbon ion irradiation: immunomodulatory effects exerted on murine tumor cell lines

**DOI:** 10.1038/s41598-020-78577-8

**Published:** 2020-12-09

**Authors:** Laura Hartmann, Philipp Schröter, Wolfram Osen, Daniel Baumann, Rienk Offringa, Mahmoud Moustafa, Rainer Will, Jürgen Debus, Stephan Brons, Stefan Rieken, Stefan B. Eichmüller

**Affiliations:** 1 German Cancer Research Center (DKFZ), Research Group GMP & T Cell Therapy, Heidelberg, Germany; 2 Faculty of Biosciences, Heidelberg University, Heidelberg, Germany; 3 Department of Radiation Oncology, Heidelberg University Hospital (UKHD), Heidelberg, Germany; 4 Heidelberg Institute of Radiation Oncology (HIRO), Heidelberg, Germany; 5 Department of Radiation Oncology, Heidelberg Ion-Beam Therapy Center (HIT), Heidelberg University Hospital (UKHD), Heidelberg, Germany; 6 German Cancer Research Center (DKFZ), Molecular Oncology of Gastrointestinal Tumors, Heidelberg, Germany; 7 Department of Surgery, Heidelberg University Hospital (UKHD), Heidelberg, Germany; 8 Faculty of Medicine Heidelberg (MFHD), Division of Molecular and Translational Radiation Oncology, Heidelberg, Germany; 9 German Cancer Consortium (DKTK) Core-Center Heidelberg, German Cancer Research Center (DKFZ), Heidelberg, Germany; 10 Department of Clinical Pathology, Suez Canal University, Ismailia, Egypt; 11 German Cancer Research Center (DKFZ), Genomics and Proteomics Core Facility, Heidelberg, Germany; 12 Department of Radiation Oncology, University Medical Center Göttingen, Göttingen, Germany

**Keywords:** Radiotherapy, Pancreatic cancer, Breast cancer, Tumour immunology

## Abstract

While for photon radiation hypofractionation has been reported to induce enhanced immunomodulatory effects, little is known about the immunomodulatory potential of carbon ion radiotherapy (CIRT). We thus compared the radio-immunogenic effects of photon and carbon ion irradiation on two murine cancer cell lines of different tumor entities. We first calculated the biological equivalent doses of carbon ions corresponding to photon doses of 1, 3, 5, and 10 Gy of the murine breast cancer cell line EO771 and the OVA-expressing pancreatic cancer cell line PDA30364/OVA by clonogenic survival assays. We compared the potential of photon and carbon ion radiation to induce cell cycle arrest, altered surface expression of immunomodulatory molecules and changes in the susceptibility of cancer cells to cytotoxic T cell (CTL) mediated killing. Irradiation induced a dose-dependent G2/M arrest in both cell lines irrespective from the irradiation source applied. Likewise, surface expression of the immunomodulatory molecules PD-L1, CD73, H2-D^b^ and H2-K^b^ was increased in a dose-dependent manner. Both radiation modalities enhanced the susceptibility of tumor cells to CTL lysis, which was more pronounced in EO771/Luci/OVA cells than in PDA30364/OVA cells. Overall, compared to photon radiation, the effects of carbon ion radiation appeared to be enhanced at higher dose range for EO771 cells and extenuated at lower dose range for PDA30364/OVA cells. Our data show for the first time that equivalent doses of carbon ion and photon irradiation exert similar immunomodulating effects on the cell lines of both tumor entities, highlighted by an enhanced susceptibility to CTL mediated cytolysis in vitro.

## Introduction

The immunomodulatory potential of radiotherapy, especially when applied in combination with immunological checkpoint inhibitors, has opened new perspectives for systemic treatment strategies against cancerous disease^1^. Although the underlying mechanisms have not been elucidated yet, there is now consensus that radiotherapy can act as a radiogenic in situ vaccine^2^ capable of inducing immunogenic cell death^3–5^ and immunogenic modulation^6,7^.

While there is evidence that low dose irradiation can promote formation of a pro-immunogenic tumor environment^8^, several preclinical investigations have suggested hypofractionation with subablative doses up to 8 Gy^9–13^, as well as ablative single doses up to 60 Gy^11,14–16^, to be most effective in inducing inflammatory stimuli and concomitant immune responses, particularly when combined with antibody administration against PD-L1 and/or CTLA-4^17^.

However, little is known about the immunomodulatory efficacy of particle radiation, such as carbon ion radiotherapy (CIRT). With respect to carbon ion and proton irradiation, reports stating increased^18,19^ or comparable^20^ immune stimulating properties have been published. While the question whether the high linear energy transfer (LET) of carbon ion radiation correlates with its immunomodulatory potential is still under debate, the increased dose conformity and independency from tumor oxygenation represent indisputable advantages of this modality, especially for treatment of hypoxic tumor entities such as breast cancer^21^ and pancreatic ductal adenocarcinoma (PDA)^22,23^.

Here, we directly compare the immunomodulatory effects of photon and carbon ion irradiation on the murine breast cancer line EO771. Moreover, we analyzed carbon ion irradiation-mediated immunomodulatory effects on the pancreatic cancer cell line PDA30364/OVA^24^ and compared these effects to photon radiation induced alterations in this cell line published previously by us^25^. For this purpose, physical single doses of 0.12, 1.11, 3.08, and 8.0 Gy and 0.1, 0.4, 1.0 and 3.1 Gy carbon ions, for EO771 and PDA30364/OVA, respectively, determined as biologically equivalent to 1, 3, 5, and 10 Gy photon radiation by clonogenic survival assays, were applied to both cell lines. The resulting impact on immunological phenotype and function of the irradiated tumor cells was subsequently investigated.

## Results

### Clonogenic survival after photon and carbon ion irradiation, calculation of relative biological effectiveness (RBE), and impact on irradiation-induced cell death

**Figure 1:**
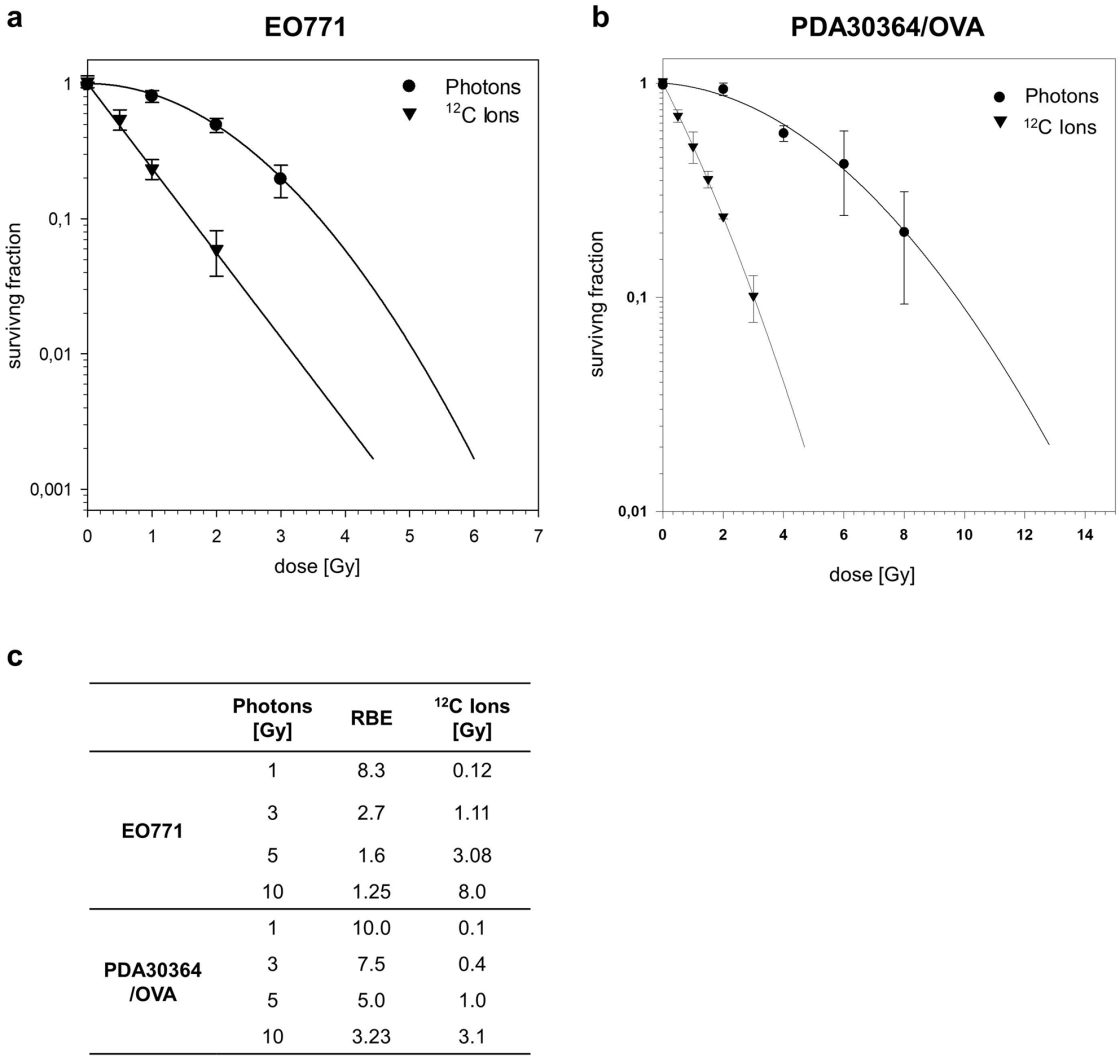
Radiation survival curves and RBE calculation for EO771 and PDA30364/OVA cells in response to photon or carbon ion irradiation. Clonogenic survival assays applying graded doses of photon or carbon ion irradiation to EO771 cells (**a**) and PDA30364/OVA cells (**b**). Mean values ± SD of triplicates from one (EO771) or two (PDA30364/OVA) independent experiments are shown. Physical doses of carbon ions biologically defined as equivalent to 1, 3, 5 and 10 Gy photon radiation applying dose-dependent RBEs are shown in (**c**).

We first compared the impact of carbon ion vs. photon irradiation on clonogenic survival of the breast cancer cell line EO771 and PDA30364/OVA cells, respectively. Both cell lines were irradiated with single doses ranging from 1.0 to 8.0 Gy photons or 0.5 to 3.0 Gy carbon ions and surviving fractions were used to perform a linear quadratic fit (LQ-fit) (Fig. 1a,b). Irradiation with carbon ions abrogated clonogenicity of EO771 and PDA30364/OVA cells with higher efficiency compared to photon irradiation resulting in a steeper dose–response relationship depicted by the almost linear slope of survival curves for carbon ion irradiation (Fig. 1a,b).

We next investigated differences in the biological effects of photon and carbon ion radiation for various biological endpoints applying radiation doses of carbon ions corrected for enhanced relative biological effectiveness (RBE). Therefore, we calculated biologically equivalent doses (BED) of carbon ion radiation based on clonogenic survival to single photon doses of 1, 3, 5 and 10 Gy. As RBE represents a dose-dependent parameter, we applied RBE values ranging from 8.3 to 1.25 resulting in photon-equivalent physical doses of 0.12, 1.11, 3.08 and 8.0 Gy carbon ions in the case of EO771 cells (Fig. 1c, top). Accordingly, for PDA30364/OVA cells we applied RBE values ranging from 10 to 3.23 resulting in photon-equivalent physical doses of 0.1, 0.4, 1.0 and 3.1 Gy carbon ions (Fig. 1c, bottom). Throughout the manuscript, we refer to physical doses of carbon ions.

To further assess irradiation-induced cytotoxicity, we investigated alterations in cell death by flow cytometric analysis of the apoptosis/necrosis markers Annexin V/ 7-AAD (Supplementary Fig. S1). Regarding EO771 cells, the overall impact of photon and carbon ion irradiation on the induction of early apoptosis (Annexin V^+^ 7-AAD^−^), and late apoptosis/necrosis (Annexin V^+^ 7-AAD^+^) was comparable. Thus, for both irradiation modalities, no major cytotoxic effects were observed 12 h after irradiation, while the proportion of both early apoptotic and late apoptotic/necrotic cells increased in a dose-dependent manner overtime. In contrast, PDA30364/OVA cells were more resistant to irradiation-induced apoptosis/necrosis after photon irradiation. Here, only the highest dose of 10 Gy resulted in an increased proportion of late apoptotic/necrotic cells 60 h after irradiation, whereas the fraction of early apoptotic cells was marginal. Regarding doses < 10 Gy greater than 90% of cells were viable at all time points tested.

### Cell cycle analysis following irradiation with photons and carbon ions

In the following step, we analyzed the cell cycle patterns of EO771 and PDA30364/OVA cells following irradiation with photons or carbon ions. While the results of carbon ion irradiation on PDA30364/OVA cells are presented below, respective effects caused by photon irradiation on this cell line have been described previously by us^25^.

**Figure 2:**
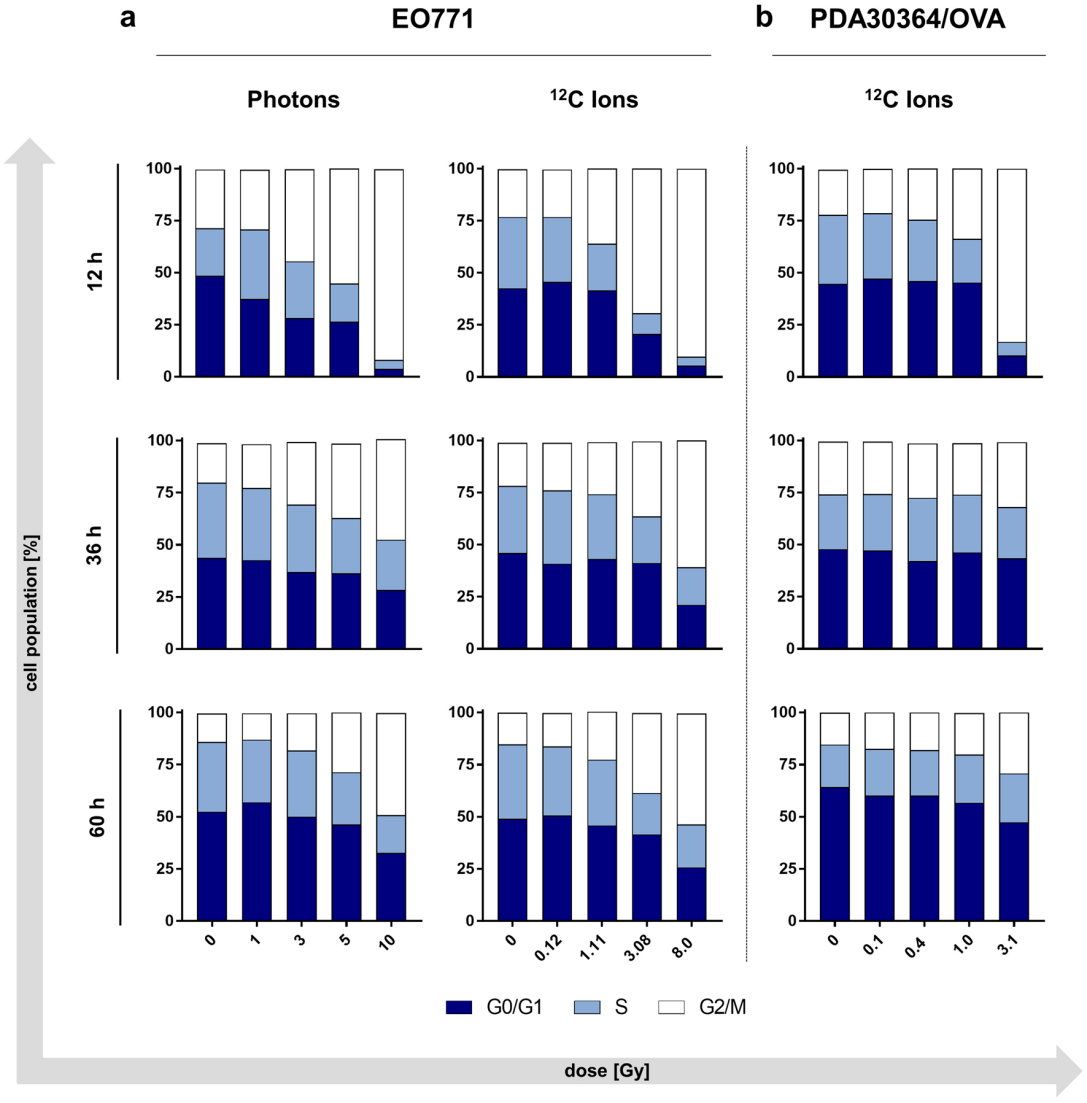
Cell cycle analysis of EO771 and PDA30364/OVA cells after photon or carbon ion irradiation. Quantification of cell cycle stages within EO771 cells (**a**) and PDA30364/OVA cells (**b**) 12, 36 and 60 h after irradiation with photons (**a**, left and Ref.^25^) or carbon ions (**a**, right and **b**) biologically equivalent to the indicated photon doses. DNA content was determined by propidium iodide staining followed by flow cytometric analysis. Representative results of one out of two independent experiments performed are presented.

Irradiation of EO771 cells with high doses, i.e. 5 or 10 Gy photons and 3.08 or 8.0 Gy carbon ions, resulted in a high proportion of cells in G2/M phase 12 h after treatment, which declined again at later time points. This transient accumulation of cells in G2/M phase appeared irrespective of the radiation modality (Fig. 2a left and middle column).

Similarly to EO771 cells, alterations in cell cycle composition of PDA30364/OVA cells in response to carbon ion irradiation were dominated by a transient and dose-dependent accumulation of cells in G2/M phase (Fig. 2b). However, compared to the previously reported response of PDA30364/OVA cells towards single photon doses, the extent of G2/M cell cycle arrest induction after 12 h following carbon ion irradiation appeared to be reduced for doses < 3.1 Gy carbon ions compared to equivalent doses of photon radiation < 10 Gy^25^. Taken together, for PDA30364/OVA we observed a differential effect of photons and carbon ions on induction of cell cycle arrest in G2/M phase at lower irradiation doses after 12 h. This did not apply to EO771 cells for which alterations of the cell cycle composition following irradiation with biologically equivalent doses of carbon ions and photons appeared to be comparable with respect to type and temporal dynamic of cell cycle arrest induced. Histograms showing cell cycle analyses are depicted in Supplementary Fig. S2.

### Photon and carbon ion irradiation induce enhanced expression of immunomodulatory cell surface molecules

We next analyzed the effect of increasing irradiation doses on the expression of immunomodulatory cell surface molecules by EO771 and PDA30364/OVA cells. Therefore, surface expression of the immune checkpoint molecules PD-L1 and CD73, involved in T cell death and suppression, respectively, and of MHC I molecules was determined by flow cytometry following irradiation.

**Figure 3:**
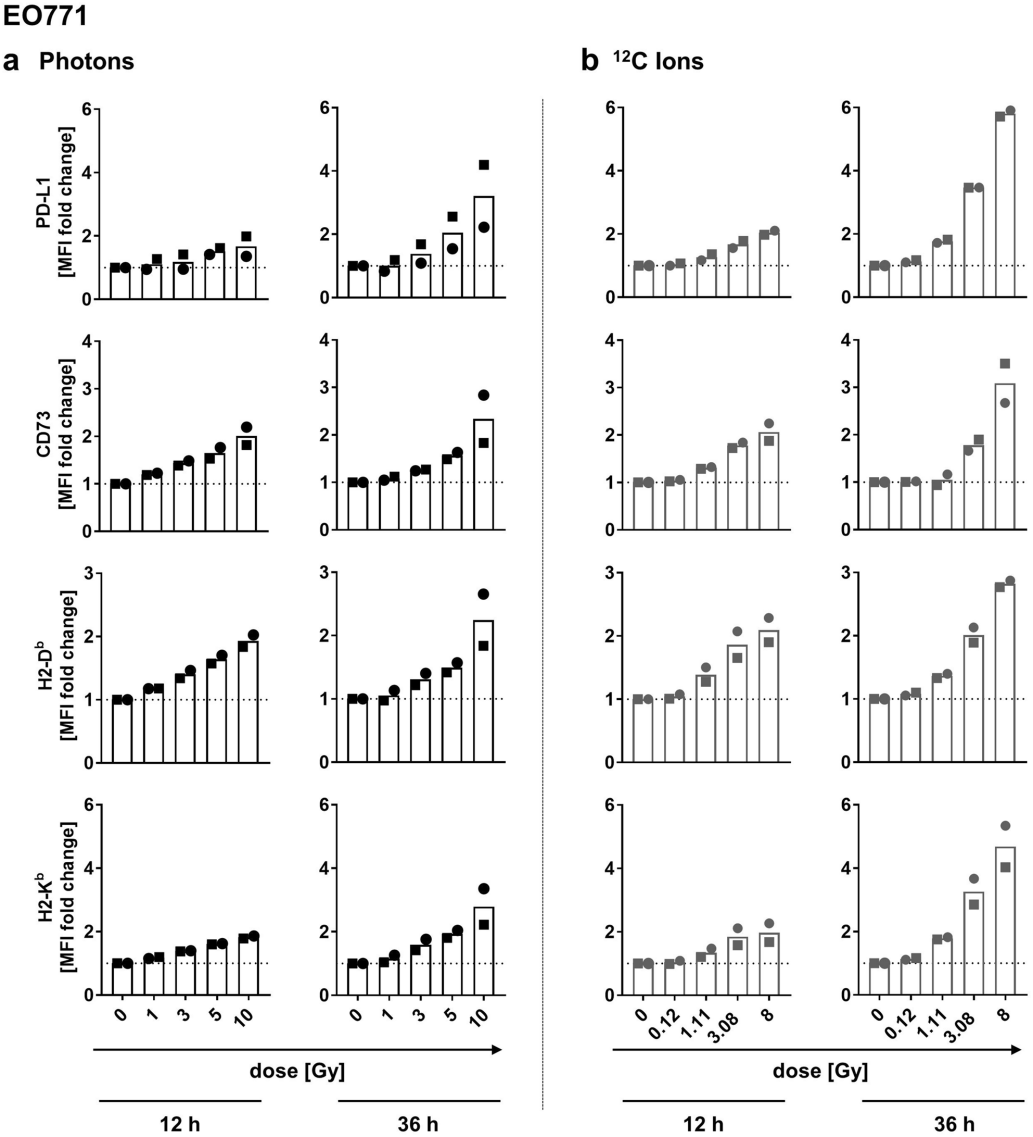
Cell surface expression of immunomodulatory molecules on EO771 cells irradiated with photons or carbon ions. Flow cytometric analysis of PD-L1, CD73 and MHC-I cell surface expression on EO771 cells 12 and 36 h after irradiation with graded doses of photons (**a**) or biologically equivalent doses of carbon ions (**b**). Depicted are fold changes of MFI (mean fluorescence intensity) normalized to MFI of non-irradiated cells. At least 40,000 viable cells were acquired per sample. Results of two experiments performed are shown (squares: experiment 1; dots: experiment 2).

Applying biologically equivalent doses of photons and carbon ions, we observed a dose-dependent increase in expression of PD-L1, CD73 and both MHC I isotypes H2-D^b^ and H2-K^b^ on the surface of EO771 cells within 12 h after irradiation, becoming even further enhanced after 36 h (Fig. 3a,b). This dose-dependent increase of cell surface expression was observed for all immunomodulatory molecules analyzed and appeared in line with the gene expression profiles determined 36 h after irradiation, showing a dose-dependent increase of immunomodulatory molecule expression also on transcriptional level (Supplementary Fig. S3). Interestingly, in both experiments the magnitude of increase in PD-L1 expression of EO771 cells was greater upon carbon ion irradiation compared to treatment with biologically equivalent doses of photons when analyzed by flow cytometry 36 h after irradiation (Fig. 3a,b).

Regarding PDA30364/OVA cells, increased surface expression of PD-L1 and CD73 molecules was detected upon irradiation with 1.0 Gy and 3.1 Gy carbon ions (Supplementary Fig. S4), equivalent to 5 and 10 Gy photon irradiation. However, surface expression levels of H2-D^b^ and H2-K^b^ molecules were hardly affected, even at high doses (Supplementary Fig. S4), which differs from the results observed with EO771 cells (Fig. 3b) and from the small increase of H2-D^b^ expression observed after photon irradiation published before^25^. Overall, the radiogenic alterations in expression levels of immunomodulatory molecules by PDA30364/OVA cells after carbon ion irradiation were less pronounced at doses < 3.1 Gy carbon ions compared to the equivalent doses of photon irradiation < 10 Gy^25^.

### Irradiation sensitizes tumor cells to CTL mediated cytolysis

**Figure 4:**
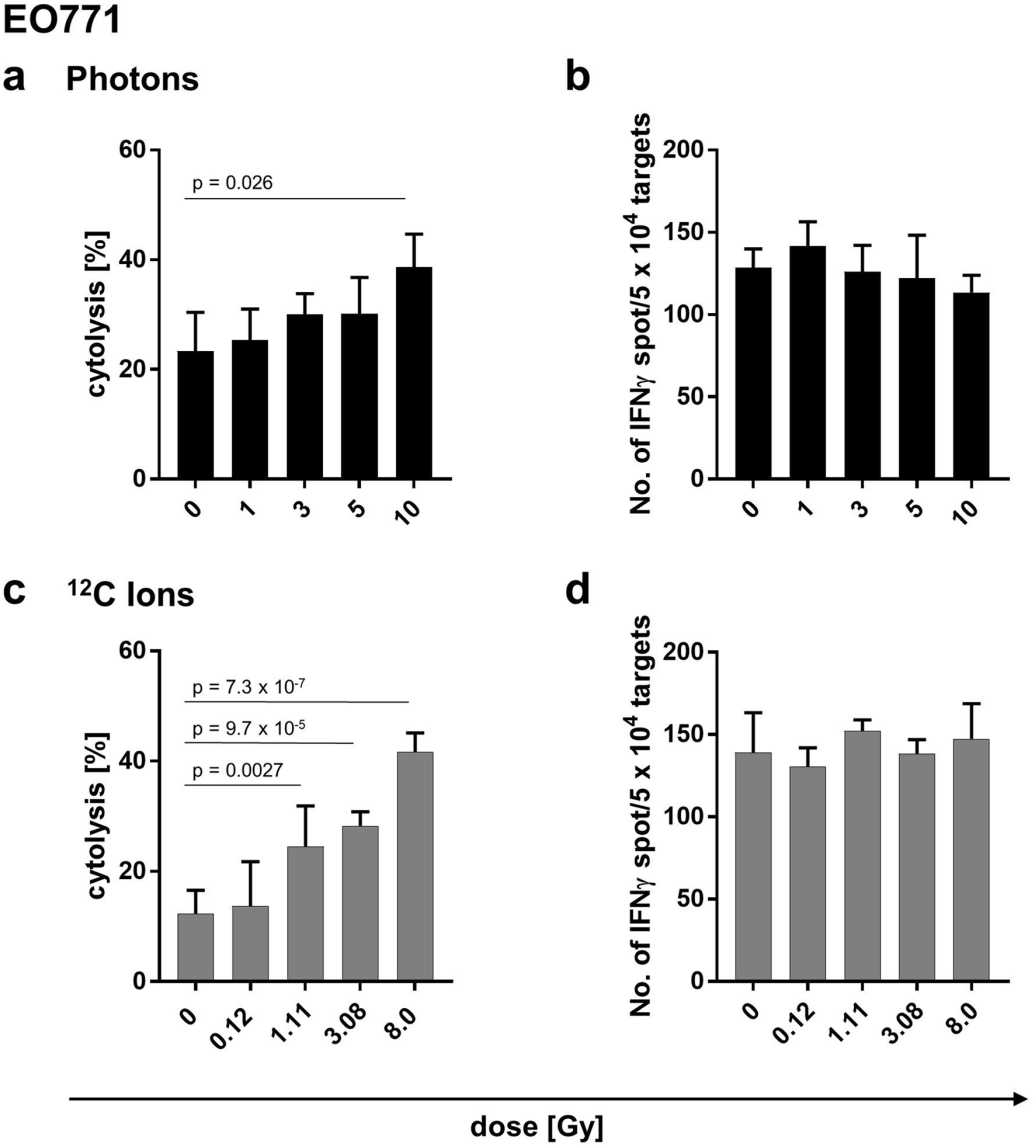
Irradiation enhances susceptibility of EO771/Luci/OVA cells to CTL recognition. Irradiation of EO771/Luci/OVA cells with increasing doses of photons (**a**) or carbon ions (**c**) enhanced cytolysis of target cells as measured by luciferase-based cytotoxicity assay, but did not affect IFNγ secretion of OVA-specific CTLs in IFNγ ELISpot assays (**b**,**d**). Representative results of one out of three (photons) and two (carbon ions) independent experiments performed are shown.

Prompted by the observation that irradiation boosted the expression of immunomodulatory molecules in both cell lines, we tested whether these radiogenic alterations affected the susceptibility of irradiated tumor cells to recognition by cytotoxic T cells. Thus, EO771/Luci/OVA cells co-expressing luciferase and OVA were co-cultured with an established OVA-specific CTL line and lack of luciferase activity was determined as measure of cytotoxicity^26^. We found that biologically equivalent doses of photon and carbon ion irradiation enhanced susceptibility of EO771/Luci/OVA cells to CTL lysis in a dose-dependent manner (Fig. 4a,c and Supplementary Fig. S5a,b). Interestingly, while CTL susceptibility of irradiated target cells was enhanced, the capacity of IFNγ secretion by the responding CTL line remained unaffected (Fig. 4b,d). Irradiation induced effects on target cell viability per se were only marginal (Supplementary Fig. S5c).

**Figure 5:**
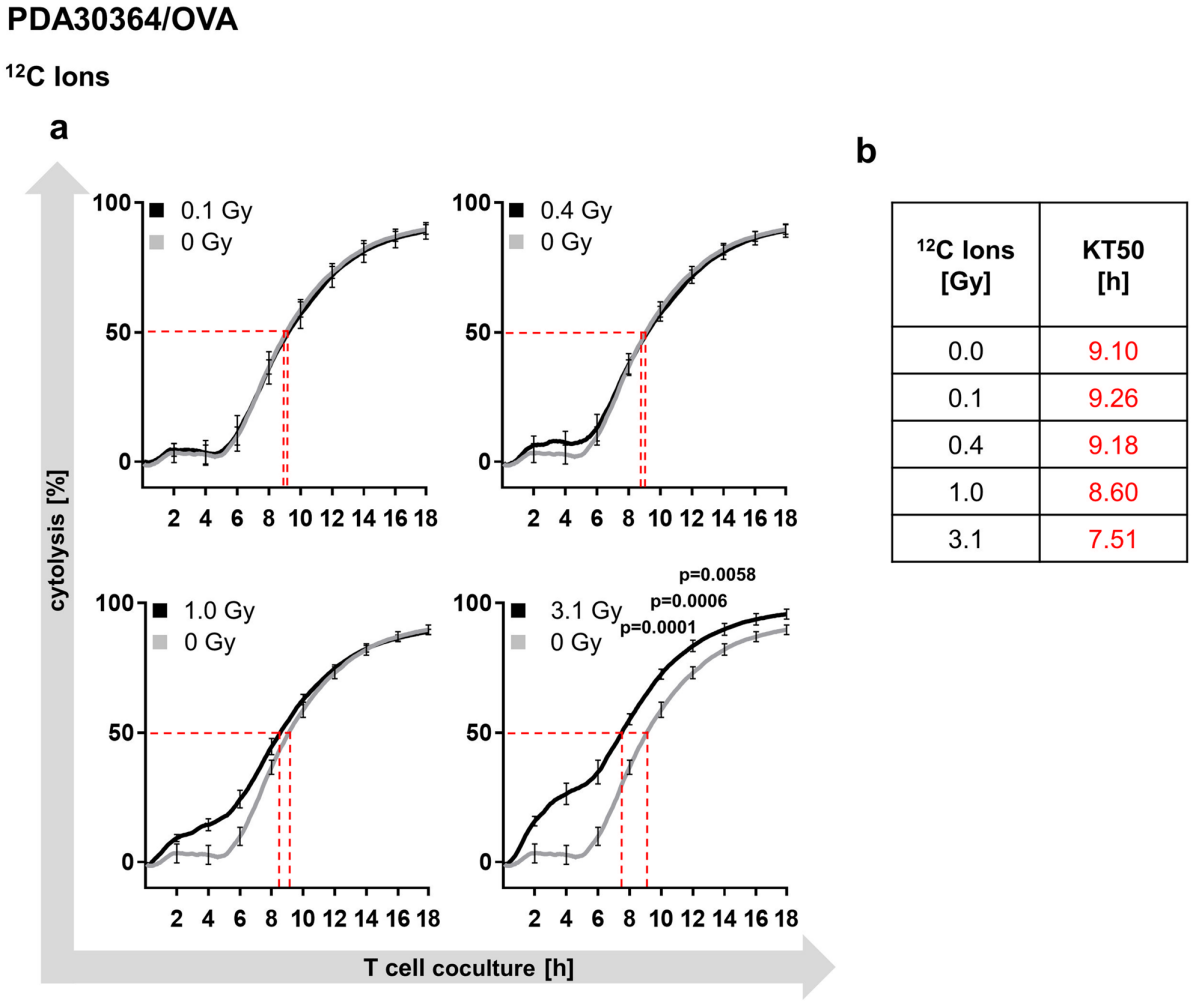
Carbon ion irradiation enhances susceptibility of PDA30364/OVA cells to CTL lysis. Cytolysis of PDA30364/OVA cells following irradiation with increasing carbon ion doses monitored for 18 h and quantified by impedance-based cytotoxicity assay (xCELLigence); dashed lines show “Kill-Time-50” (KT50) values; effector to target cell ratio was 2.5:1 (**a**). Time span elapsed until 50% of target cells had underwent CTL mediated lysis was expressed as KT50 (**b**). Representative results of one out of three independent experiments performed are shown.

We have previously shown that single dose photon irradiation leads to a dose-dependent increase in susceptibility of PDA30364/OVA cells to CTL mediated lysis^25^. In the same way, we now investigated the susceptibility of carbon ion irradiated PDA30364/OVA cells to OVA-specific CTL lysis through impedance-based cytotoxicity assays (xCELLigence assay). Applying biologically equivalent doses of carbon ions, we found that irradiation induced enhanced killing of target cells in a dose-dependent manner, resulting in earlier onset of cytolysis of irradiated cells compared to non-irradiated control. This effect became evident upon irradiation with 0.4 Gy carbon ions and increased with higher doses (Fig. 5a). In fact, irradiation with 3.1 Gy carbon ions, which is equivalent to 10 Gy photons, resulted in a significant increase in cytolysis compared to untreated cells persisting for 18 h following CTL co-culture (Fig. 5a).

Further, we determined the time span required by CTLs to kill 50% of irradiated target cells, expressed as “Kill-Time-50 (KT50)”. Compared to control (0 Gy), a reduction in KT50 was observed at higher irradiation doses of 1.0 and 3.1 Gy carbon ions (Fig. 5b) equivalent to 5 and 10 Gy photons, respectively. The extent of KT50 reduction relative to the non-irradiated control was of comparable magnitude for both maximal equivalent doses, with 19.8% for 10 Gy photons described previously^25^ and 17.5% for 3.1 Gy carbon ions (Fig. 5b). Overall, radiogenic sensitization of PDA30364/OVA towards CTL mediated lysis followed, again, the same pattern as observed after photon irradiation^25^.

The CTL line employed expresses PD-1^25^ enabling target cell interaction via the PD-1/PD-L1 axis. Having observed a moderate, yet dose-dependent increase in cell surface expression of PD-L1 by PDA30364/OVA cells after photon^25^ and carbon ion irradiation (Supplementary Fig. S4), we tested for both radiation types whether addition of PD-L1-blocking antibody would increase tumor cell specific lysis in synergism to irradiation (Supplementary Fig. S6). Overall, addition of PD-L1-blocking antibody showed no direct synergism to irradiation with respect to enhanced CTL mediated killing in this particular in vitro setting for PDA30364/OVA.

## Discussion

In the present study, we compared radiogenic effects exerted on two murine cancer cell lines by photon and carbon ion irradiation. Irradiation with heavy ions, such as carbon ions, shows steep dose gradients with high energy losses along the way of the primary beam (high LET), resulting in a higher number of ionizations per unit distance covered and in an increased number of direct DNA damages compared to photon radiation. As a result, multiple DNA double-strand breaks (DSBs) are caused in close proximity, termed clustered DNA damage, which differs from the rather dispersed DSB pattern generated by conventional photon irradiation^27^. The enhanced efficacy of DNA damage induction results in the well-known superior capacity of carbon ion irradiation to induce anti-proliferative effects that are primarily linked to lethal chromosomal aberrations. However, it is still a matter of debate whether the high-LET properties of carbon ion irradiation induce phenotypic alterations among tumor cells that result in radiogenic immunomodulation effects actually different from those caused by photons.

We demonstrated enhanced efficacy of carbon ion radiation in the abrogation of clonogenic survival of EO771 and PDA30364/OVA cells and determined dose-dependent RBEs of carbon ion radiation for both tumor cell lines. Conventionally, the biological endpoint used to define the RBE is the test radiation’s ability to impair clonogenicity of tumor cells. However, for a given cell line and test radiation, the RBE itself is not a constant but represents a dose-dependent variable and a radiobiological concept depending on additional factors such as the measured biological endpoint itself, dose-rate and beam quality as well as oxygen concentration and cell cycle phase^28,29^. In clinical application of CIRT, calculation of photon-equivalent carbon ion doses is primarily based on empirical data and experience from normofractionated photon irradiation schemes, therefore the RBE of carbon ion radiation is commonly reported to be approximately 2- to 3-fold greater than the RBE of photon radiation^30^. However, as radiogenic immunomodulation of tumor cells by conventional photon radiation is heavily dependent on dose and fractionation^17^, we strictly applied experimental dose-dependent RBEs to calculate physical carbon ion doses closely matching to photon doses of 1, 3, 5 and 10 Gy. The BEDs thus defined were consistently used throughout the experiments.

Overall, the breast cancer cell line EO771 showed higher susceptibility to radiation-induced cytotoxicity compared to the pancreatic cancer cell line PDA30364/OVA, which is also indicated by the differing surviving fractions at 2 Gy photon radiation (SF2) of 0.49 and 0.87, for EO771 and PDA30364/OVA cells, respectively. These results reflect the clinical situation, where irradiation is part of the standard therapy against breast cancer, due to the radiosensitivity of this tumor entity^31^, while the indication of radiotherapy for the treatment of PDA patients is still under debate^32–34^. The radioresistant phenotype of PDA has been found to severely complicate successful radiotherapy and multiple mechanisms have been reported to be involved^23^. The PDA cell line investigated here exhibits two of the most common driver mutations in PDA^35,36^, which are activating KRAS and p53 loss of function mutations^24^, both being reported to contribute to radioresistance of PDA via, for example, inactivation of the G1/S cell cycle checkpoint and dysregulation of apoptotic pathways^23^. The latter is reflected by our data, as doses < 10 Gy did not affect apoptosis and irradiation with 10 Gy only slightly increased the proportion of late apoptotic/necrotic cells 60 h after treatment.

Our cell cycle analyses showed a dose-dependent G2/M cell cycle arrest in EO771 cells in response to photon irradiation, similarly as seen before for PDA30364/OVA tumor cells^25^. High-LET carbon ion irradiation led to similar results with respect to quality and temporal persistence of cell cycle arrest induced. Although the G2/M arrest was of comparable magnitude for both irradiation modalities and in both tumor cell lines, a slightly milder effect in magnitude was observable in PDA30364/OVA cells upon carbon ion irradiation at doses < 3.1 Gy at the 12 h time point compared to the equivalent doses of photon irradiation < 10 Gy. These findings were not seen in EO771 cells and differ from reports stating generally more pronounced delays in S- and G2-phase with increasing LET for glioblastoma, fibroblasts and cervical cancer cells in vitro. Importantly however, most reports did not compare dose-dependent RBE-corrected biological equivalent doses of photon and carbon ion irradiation but physical doses^37–40^.

It is suspected that the high-LET properties of particle radiation such as carbon ion radiation might impact their immunomodulatory potential regarding enhanced inflammatory responses and induction of abscopal effects^30,41^. In fact, we found that irradiation with both, carbon ions and photons, enhanced the expression of immunomodulatory cell surface molecules and increased the susceptibility of tumor cells to antigen-specific CTL lysis of both tumor cell lines. Our data are partially in line with a study describing a common immunogenic modulation signature of photon and proton irradiation in vitro. In this study, a variety of human cancer cell lines showed enhanced expression of cell surface molecules involved in immune recognition as well as increased susceptibility to CTL mediated lysis induced by both radiation modalities^20^. Interestingly, in our cytotoxicity assays, the susceptibility of irradiated EO771/Luci/OVA target cells to CTL mediated lysis was enhanced, while the IFNγ secretion capacity of the responding CTL line remained unaffected, thus showing that increased target cell killing was due to enhanced susceptibility of irradiated target cells rather than caused by increased effector function of the CTL line. In fact, it has been shown that CTLs require higher levels of T cell receptor (TCR) occupancy for IFN-γ secretion than for cytolysis^42^. Thus, increased H2-K^b^ expression levels induced by irradiation might have enhanced the target cells’ susceptibility to CTL lysis without affecting IFN-γ secretion levels as a result of lower responsiveness to increased TCR occupancy, thereby possibly explaining the functional dichotomy of the CTL line used.

Recently, using human bone osteosarcoma, non-small-cell lung cancer (NSCLC) and prostate cancer cell lines in vitro, Sato et al. have shown for the first time that an increase in PD-L1 expression can emerge as a direct consequence of radiation induced DNA DSBs which was regulated by ATM/ATR/Chk1 kinases, the key enzymes involved in DNA damage signaling^43^. We noted a moderate dose-dependent upregulation of PD-L1 expression on the cell surface of both tumor cell lines following irradiation with both radiation sources. Yet, addition of PD-L1 blocking antibody did not synergize with the radiation-induced increase of CTL mediated tumor cell lysis. This might be due to the fact that the CTL line applied was maximally activated on the day of assay, thereby circumventing further activation by PD-1/PD-L1 blockade. In fact, irradiation-induced upregulation of PD-L1 expression by various cell types within the tumor microenvironment has been described and radiotherapy in combination with anti-PD-L1 treatment was shown to act in concert in normalizing the immunosuppressive tumor milieu, thus highlighting the biological significance of PD-L1 upregulation in the tumor microenvironment in response to irradiation^44^.

Local irradiation affects all cell types present in the tumor microenvironment including tumor resident T cells. Interestingly, in the murine Panc02 model, preexisting intratumoral T cells have been recently demonstrated to survive even high irradiation doses of 20 Gy. Moreover, these CTLs, although showing reduced proliferative potential, retained their motility and, most importantly, exhibited enhanced tumor antigen specific functionality in vitro and in vivo^45^*.* This appears of relevance not only for the design of clinical radio immunotherapy approaches, but also for studies investigating irradiation induced abscopal effects, where local tumor irradiation results in immune cell mediated regression of untreated tumors at distant sites. Indeed, carbon ion therapy enhanced the efficacy of immune checkpoint inhibition both on a primary and a distant tumor as recently shown in a murine osteosarcoma model^46^.

Our study shows that irradiation with photons and carbon ions shared a common profile with respect to induced cell cycle arrest, induction of increased surface expression of immunomodulating molecules and enhanced susceptibility to antigen-specific CTL mediated killing in vitro. Compared to photon radiation, RT with particles has several biophysical advantages^28,47^: potential reduction of tumor metastatic spread^48,49^ and distant metastases^46^, anti-angiogenic effects^50^ as well as reduced oxygen enhancement ratio increasing its efficacy for hypoxic tumors such as PDA^22,28^. Further studies are needed in order to investigate the immune stimulatory potential of carbon ions in vivo with the help of suitable animal tumor models.

## Materials and methods

### Cell lines and in vitro culture

The breast cancer cell line EO771 was cultured in RPMI 1640 (Thermo Fisher Scientific, Waltham, USA) containing 10% heat-inactivated FCS, 10 mmol/l HEPES (Thermo Fisher Scientific), 100 U/ml penicillin (Thermo Fisher Scientific) and 100 μg/ml streptomycin (Thermo Fisher Scientific). EO771/Luci/OVA cells were cultured in the same medium supplemented with 0.2 mg/ml geniticin (Thermo Fisher Scientific) and 1 µg/ml puromycin (Thermo Fisher Scientific). PDA30364 is a pancreatic adenocarcinoma cell line derived from PDA GEMM Elas-tTA/TetOCre Kras^+/G12D^ p53^+/R172H^ transgenic mice^51–53^. The PDA30364 derived transfectant clone expressing chicken ovalbumin (OVA) has been described previously^24^. PDA30364/OVA cells were cultured in DMEM (Thermo Fisher Scientific, Dreieich, Germany) containing 10% heat-inactivated FCS, 1 mmol/l sodium pyruvate (Thermo Fisher Scientific), 100 U/ml penicillin and 100 μg/ml streptomycin, and 10 μg/ml blasticidin S HCL (Thermo Fisher Scientific). Parental PDA30364 cells were cultured in the same medium, but without blasticidin.

The OVA-specific CTL line recognizing the H2-K^b^-restricted epitope OVA 257–264 (SIINFEKL)^54^ was cultured in alpha MEM (Sigma-Aldrich, St. Louis, USA) supplemented with 10% heat-inactivated FCS, 2.5% (v/v) supernatant of concanavalin A stimulated rat spleen cell cultures, 12.5 mmol/l methyl-α-d-mannopyranoside (Thermo Fisher Scientific), 100 U/ml penicillin, 100 μg/ml streptomycin, 2 mmol/l l-Glutamine (Thermo Fisher Scientific) and 0.1% 2-mercaptoethanol (Thermo Fisher Scientific). CTLs were expanded in 24-well plates by weekly restimulation as described^54^. All cell lines were cultured at 37 °C/5% CO_2_.

### Lentiviral transduction of EO771 cells

Transduction of EO771 cells was performed by the Genomics and Proteomics Core Facility of the DKFZ using a retroviral construct expressing red firefly luciferase and a puromycin selection marker under control of a SV40 promoter, similarly to the protocol described before ^55^. Retroviral particles were produced by co-transduction of HEK293FT (Thermo Fisher Scientific) cells with pBabe-Puro red firely luciferase expression vector and the packaging plasmids pHIT60 and pMD2G (Addgene, Middlesex, UK). Two days later, virus-containing supernatants were collected and cleared by centrifugation. After the supernatants were passed through a 0.45 μm filter, EO771 cells were transduced with viral particles at 70% confluency in the presence of 10 μg/ml polybrene (Merck KGaA, Darmstadt, Germany). Selection was started one day post transduction and clones were picked for expansion two weeks later. The OVA-encoding nucleotide sequence (RefSeq NM_205152.2.) flanked by attL recombination sites was synthesized and cloned into a pMX plasmid (Thermo Fisher Scientific). The sequences were shuttled into lentiviral expression vectors adding a C-terminal IRES sequence coupled to a neomycin resistance gene by gateway recombination. For the generation of lentiviral particles, HEK293FT cells were transduced with the lentiviral OVA expression constructs and transfected with 2nd generation viral packaging plasmids VSV.G (Addgene) and psPAX2 (Addgene). After 2 days, viral particles were purified and EO771/Luci cells transduced as described before. The clone used in this study is designated EO771/Luci/OVA.

### Photon radiotherapy

Photon irradiation was performed with a biological cabinet X-ray irradiator XRAD 320 (Precision X-ray Inc., N. Branford, USA) with a dose rate of 0.96 Gy/min or a Gammacell 40 Exactor (Best Theratronics, Ottawa, Canada) with a dose rate of 0.91 Gy/min.

### Carbon ion radiotherapy

Carbon ion radiotherapy was performed at the Heidelberg Ion-Beam Therapy Center (HIT) with the horizontal beamline using the raster scanning technique developed by Haberer et al*.*^56^. Irradiation was delivered within an 8 mm wide extended Bragg peak (dose average linear energy transfer (LET), 103 keV/μm) located at a depth of 35 mm. Cell monolayers were positioned in the middle of the extended Bragg peak using a 30 mm wide acrylic absorber.

### Clonogenic survival assays

Clonogenic survival assays were performed as outlined previously^25,57,58^. Tumor cells were irradiated with increasing doses of photons and carbon ions as depicted in Fig. 1. After 18 h of incubation, PDA30364/OVA cells were seeded into 96-well plates in triplicates adding 1 or 3 cells per well. For EO771 cells, triplicates of wells containing 1 or 2 irradiated cells, respectively, were prepared. After 14 days, colonies where fixed with 70% ethanol followed by staining with 0.2% methylene blue (Merck KGaA) for 10 min. Colonies were counted under the microscope applying a minimal threshold number of 50 cells for a colony to be considered surviving. In this format, the plating efficiency (PE) is defined by PE = 1/N × ln(96/n−): where N gives number of cells seeded per 96-well plate and n− represents the number of colony-negative wells per 96-well plate. Appropriate N was defined in previous tests ranging between 1 and 3 cells. Cellular surviving fractions (SF) were calculated according to the formula: SF = PE_treatment_/PE_control_. Survival curves and dose-dependent relative biological effectiveness (RBE) of carbon ion irradiation were modeled according to the linear-quadratic model using Sigma Plot version 12.5 (SyStat Software, San Jose, USA).

### Flow cytometry

Immunofluorescence staining was performed as described before^25^ using monoclonal antibodies shown in Supplementary Table S1. Twelve and 36 h after irradiation, cells were harvested and washed with PBS (Sigma-Aldrich, St. Louis, USA) followed by incubation with Zombie Violet™ Fixable Viability dye (1:1000) (Biolegend, San Diego, USA) or LIVE/DEAD™ Fixable Yellow Dead Cell Stain Kit (1:1000) (Thermo Fisher Scientific) in a total volume of 100 μl PBS at 4 °C for 20 min. Subsequently, cells were incubated with fluorochrome-conjugated antibodies diluted in a total volume of 100 μl PBS (2 μg/ml) containing 10% FCS or 5 μg/ml BSA (Sigma-Aldrich) and 2 mmol/l EDTA (Sigma-Aldrich) at 4 °C for 30 min. Respective isotype matched antibodies against irrelevant epitopes as well as fluorescence minus one (FMO) controls were included for each treatment condition. Delta median fluorescence intensity (∆MFI) was calculated for each irradiation dose and time point by subtracting the MFI of combined isotype and FMO control from the MFI values of stained samples. Acquisition was performed using a FACSCanto II or LSR Fortessa (BD Biosciences, Franklin Lakes, USA) flow cytometer run with FACS-Diva software version 6.2 (BD Biosciences). FlowJo software version 10.4.2 (Tree Star, Ashland, USA) was used to analyze at least 40,000 viable cells per sample.

### Cell cycle analysis

Cell cycle analysis was performed as described previously by us^25^. Briefly, 12, 36 and 60 h after irradiation with photons or carbon ions, cells were harvested and washed with PBS followed by permeabilization/fixation with ice-cold 70% ethanol at 4 °C for at least 24 h. Subsequently, cells were incubated with 200 µl RNAse A solution (100 µg/ml; AppliChem, Darmstadt, Germany) at room temperature for 10 min followed by staining with 5 µg propidium iodide solution (Sigma-Aldrich) at room temperature overnight. Acquisition was performed with a FACSCanto II cytometer using FACS-Diva software version 6.2 (BD Biosciences). Based on the DNA content, G0/G1-, S-, or G2/M cell cycle stages were determined. FlowJo software version 10.4.2 (Tree Star) was used to analyze at least 10,000 events per sample.

### Luciferase-based cytotoxicity assay

Irradiated EO771 cells were rested for 12 h at 37 °C. Then, 5 × 10^3^ target cells were added per well of a 96-well plate (Perkin Elmer, Waltham, USA) and co-cultured with OVA-specific CTLs at an effecter/tumor cell ratio of 1:10. After 16–20 h, supernatants including CTLs were removed and luciferase activity of remaining target cells was determined as a measure for viable target cells as described elsewhere^26^. One quadruplicate of target cells was cultured without CTLs to control for irradiation-induced cell death. Luminescence was quantified using a Mithras LB 940 Multimoad Micropate Reader (Berthold Technologies, Bad Wilsbad, Germany) collecting light for 0.2 s per well. The relative luminescence units (RLU) are proportional to the amonut of viable cells. Percentage cytolysis was calculated with the following formula: Cytolysis [%] = ((RLU_wo.CTLs_ − RLU_w.CTLs_)/RLU_wo.CTLs_) × 100. Standard deviation (SD) was calculated using error propagation formulas.

### IFNγ ELISpot assay

The membrane of a MultiScreen_HTS_-IP ELISpot plate (Merck) was pre-wet with 80% ethanol for 2 min, washed twice with PBS and coated with 1 μg/ml rat anti-mouse IFNγ capture antibody (BD Biosciences) diluted in PBS overnight at 4 °C. Plates were washed twice with PBS, and blocked with culture medium for 1 h at 37 °C. 12 h after irradiation, 5 × 10^4^ irradiated EO771/Luci/OVA were co-cultured with 4 × 10^2^ OVA specific-CTLs for 16–20 h at 37 °C. Then, plates were washed five times with PBS containing 0.5% Tween 20 and once with PBS and 1 µg/ml biotinylated rat anti-mouse IFN-γ antibody (BD Biosciences) diluted in PBS was added for 1 h at 4 °C. Having washed plates four times with PBS, wells were incubated with streptavidin-conjugated alkaline phosphatase (AKP) (1:500 in PBS, BD Biosciences) for 30 min at room temperature. Again, plates were washed four times with PBS and developed with BCIP/NBT substrate (Sigma-Adrich) for 1–3 min. The colorimetric reaction was stopped by rinsing the plate with distilled water. Plates were analyzed with the CTL ELISpot Reader System running the ImmunoSpot^®^ Software (CTL Europe GmbH, Bonn, Germany).

### Real-time cytotoxicity assay

CTL lysis of PDA30364/OVA cells was assessed in vitro using the impedance-based xCELLigence Real-Time Cell Analyzer System (RTCA) (ACEA Biosciences, San Diego, USA). Eighteen h after irradiation, tumor cells were seeded into an E-Plate 96 (ACEA Biosciences) at a density of 7.2 × 10^3^ cells/well and rested overnight at 37 °C. Next day, CTLs were added at an effector/tumor cell ratio of 2.5:1. PD-L1-specific antibody (20 µg/ml) (Bio X Cell, Inc., West Lebanon, USA) was added 3 h prior to CTL addition and at the time point the co-culture was started, resulting in a final concentration of 10 μg/ml αPD-L1 antibody. The cell index (CI), representing the relative impedance as a measure for the number of adherent cells was determined every 5 min for at least 24 h. CI values were normalized to the time point of CTL addition using the RTCA Software 2.0 (ACEA Biosciences). Percentage cytolysis was calculated according to the formula: Cytolysis [%] = ((CI_wo.CTLs_ − CI_w.CTLs_)/CI_wo.CTLs_) × 100. Standard deviation (SD) of mean CI values was calculated using error propagation formulas established by the Biostatistics Department of the DKFZ. Specificity of the CTL line was controlled using parental PDA30364 cells in comparison to PDA30364/OVA cells as described previously^25^. “Kill-Time-50” (KT50) was defined as time span between CTL addition and eradication of 50% of PDA30364/OVA cells.

### Statistical analysis

For cytotoxicity assays significant differences among fractions of cytolysis obtained from unirradiated cells were compared to the ones of each irradiation dose at a given time point. Significance was determined via R software version 3.6.1 (RStudio, Boston, USA) using a two-tailed *t* test calculated with an R code created by the DKFZ Biostatistics Department. To correct for multiple comparison, we applied Holm-Bonferroni method.

## Supplementary Information

Supplementary Information.

## Data Availability

All data generated or analyzed during this study are included in this article (and its Supplementary Information files).

## References

[1] Wang Y (2018). Combining immunotherapy and radiotherapy for cancer treatment: Current challenges and future directions. Front. Pharmacol..

[2] Formenti SC, Demaria S (2012). Radiation therapy to convert the tumor into an in situ vaccine. Int. J. Radiat. Oncol. Biol. Phys..

[3] Galluzzi L (2020). Consensus guidelines for the definition, detection and interpretation of immunogenic cell death. J. Immunother. Cancer.

[4] Sia J, Szmyd R, Hau E, Gee HE (2020). Molecular mechanisms of radiation-induced cancer cell death: A primer. Front. Cell Dev. Biol..

[5] Golden EB, Pellicciotta I, Demaria S, Barcellos-Hoff MH, Formenti SC (2012). The convergence of radiation and immunogenic cell death signaling pathways. Front. Oncol..

[6] Gameiro SR (2014). Radiation-induced immunogenic modulation of tumor enhances antigen processing and calreticulin exposure, resulting in enhanced T-cell killing. Oncotarget.

[7] Gameiro SR, Ardiani A, Kwilas A, Hodge JW (2014). Radiation-induced survival responses promote immunogenic modulation to enhance immunotherapy in combinatorial regimens. Oncoimmunology.

[8] Klug F (2013). Low-dose irradiation programs macrophage differentiation to an iNOS(+)/M1 phenotype that orchestrates effective T cell immunotherapy. Cancer Cell.

[9] Dewan MZ (2009). Fractionated but not single-dose radiotherapy induces an immune-mediated abscopal effect when combined with anti-CTLA-4 antibody. Clin. Cancer Res..

[10] Tsukui H (2020). CD73 blockade enhances the local and abscopal effects of radiotherapy in a murine rectal cancer model. BMC Cancer.

[11] Twyman-Saint Victor C (2015). Radiation and dual checkpoint blockade activate non-redundant immune mechanisms in cancer. Nature.

[12] Vanpouille-Box C (2017). DNA exonuclease Trex1 regulates radiotherapy-induced tumour immunogenicity. Nat. Commun..

[13] Vanpouille-Box C (2015). TGFbeta is a master regulator of radiation therapy-induced antitumor immunity. Cancer Res..

[14] Chakravarty PK (1999). Flt3-ligand administration after radiation therapy prolongs survival in a murine model of metastatic lung cancer. Cancer Res..

[15] Lee Y (2009). Therapeutic effects of ablative radiation on local tumor require CD8+ T cells: Changing strategies for cancer treatment. Blood.

[16] Lugade AA (2005). Local radiation therapy of B16 melanoma tumors increases the generation of tumor antigen-specific effector cells that traffic to the tumor. J. Immunol..

[17] Ko EC, Benjamin KT, Formenti SC (2018). Generating antitumor immunity by targeted radiation therapy: Role of dose and fractionation. Adv. Radiat. Oncol..

[18] Shimokawa T, Ma L, Ando K, Sato K, Imai T (2016). The future of combining carbon-ion radiotherapy with immunotherapy: Evidence and progress in mouse models. Int. J. Part. Ther..

[19] Ando K (2013). Effective suppression of pulmonary metastasis in combined carbon ion radiation therapy with dendritic-cell immunotherapy in murine tumor models. Int. J. Radiat. Oncol. Biol. Phys..

[20] Gameiro SR (2016). Tumor cells surviving exposure to proton or photon radiation share a common immunogenic modulation signature, rendering them more sensitive to T cell-mediated killing. Int. J. Radiat. Oncol. Biol. Phys..

[21] Milani M, Harris AL (2008). Targeting tumour hypoxia in breast cancer. Eur J Cancer.

[22] Kamada T (2015). Carbon ion radiotherapy in Japan: An assessment of 20 years of clinical experience. Lancet Oncol..

[23] Seshacharyulu P (1868). Biological determinants of radioresistance and their remediation in pancreatic cancer. Biochim. Biophys. Acta Rev. Cancer.

[24] Baumann D (2020). Proimmunogenic impact of MEK inhibition synergizes with agonist anti-CD40 immunostimulatory antibodies in tumor therapy. Nat. Commun..

[25] Schröter P (2020). Radiation-induced alterations in immunogenicity of a murine pancreatic ductal adenocarcinoma cell line. Sci. Rep..

[26] Khandelwal N (2015). A high-throughput RNAi screen for detection of immune-checkpoint molecules that mediate tumor resistance to cytotoxic T lymphocytes. EMBO Mol. Med..

[27] Hagiwara Y (2017). 3D-structured illumination microscopy reveals clustered DNA double-strand break formation in widespread gammaH2AX foci after high LET heavy-ion particle radiation. Oncotarget.

[28] Durante M, Loeffler JS (2010). Charged particles in radiation oncology. Nat. Rev. Clin. Oncol..

[29] Schardt D, Elsasser T, Schulz-Ertner D (2010). Heavy-ion tumor therapy: Physical and radiobiological benefits. Rev. Mod. Phys..

[30] Ebner DK (2017). The immunoregulatory potential of particle radiation in cancer therapy. Front. Immunol..

[31] Castaneda SA, Strasser J (2017). Updates in the treatment of breast cancer with radiotherapy. Surg. Oncol. Clin. N. Am..

[32] Hall WA, Goodman KA (2019). Radiation therapy for pancreatic adenocarcinoma, a treatment option that must be considered in the management of a devastating malignancy. Radiat. Oncol..

[33] Versteijne E (2020). Preoperative chemoradiotherapy versus immediate surgery for resectable and borderline resectable pancreatic cancer: Results of the Dutch Randomized Phase III PREOPANC Trial. J. Clin. Oncol..

[34] Chang JS, Chiu YF, Yu JC, Chen LT, Ch'ang HJ (2018). The role of consolidation chemoradiotherapy in locally advanced pancreatic cancer receiving chemotherapy: An updated systematic review and meta-analysis. Cancer Res. Treat..

[35] Waddell N (2015). Whole genomes redefine the mutational landscape of pancreatic cancer. Nature.

[36] Jones S (2008). Core signaling pathways in human pancreatic cancers revealed by global genomic analyses. Science.

[37] Fournier C, Taucher-Scholz G (2004). Radiation induced cell cycle arrest: An overview of specific effects following high-LET exposure. Radiother. Oncol..

[38] Ghorai A, Bhattacharyya NP, Sarma A, Ghosh U (2014). Radiosensitivity and induction of apoptosis by high LET carbon ion beam and low LET gamma radiation: A comparative study. Scientifica (Cairo).

[39] Tsuboi K (2007). Cell cycle checkpoint and apoptosis induction in glioblastoma cells and fibroblasts irradiated with carbon beam. J. Radiat. Res..

[40] Tsuboi K, Tsuchida Y, Nose T, Ando K (1998). Cytotoxic effect of accelerated carbon beams on glioblastoma cell lines with p53 mutation: Clonogenic survival and cell-cycle analysis. Int. J. Radiat. Biol..

[41] Durante M, Reppingen N, Held KD (2013). Immunologically augmented cancer treatment using modern radiotherapy. Trends Mol. Med..

[42] Valitutti S, Muller S, Dessing M, Lanzavecchia A (1996). Different responses are elicited in cytotoxic T lymphocytes by different levels of T cell receptor occupancy. J. Exp. Med..

[43] Sato H (2017). DNA double-strand break repair pathway regulates PD-L1 expression in cancer cells. Nat. Commun..

[44] Deng L (2014). Irradiation and anti-PD-L1 treatment synergistically promote antitumor immunity in mice. J. Clin. Investig..

[45] Arina A (2019). Tumor-reprogrammed resident T cells resist radiation to control tumors. Nat. Commun..

[46] Takahashi Y (2019). Carbon ion irradiation enhances the antitumor efficacy of dual immune checkpoint blockade therapy both for local and distant sites in murine osteosarcoma. Oncotarget.

[47] Amaldi U, Kraft G (2005). Radiotherapy with beams of carbon ions. Rep. Prog. Phys..

[48] Lee KS, Lee DH, Chun SY, Nam KS (2014). Metastatic potential in MDA-MB-231 human breast cancer cells is inhibited by proton beam irradiation via the Akt/nuclear factor-kappaB signaling pathway. Mol. Med. Rep..

[49] Ogata T (2005). Particle irradiation suppresses metastatic potential of cancer cells. Cancer Res..

[50] Takahashi Y (2003). Heavy ion irradiation inhibits in vitro angiogenesis even at sublethal dose. Cancer Res..

[51] Guerra C (2007). Chronic pancreatitis is essential for induction of pancreatic ductal adenocarcinoma by K-Ras oncogenes in adult mice. Cancer Cell.

[52] Jackson EL (2001). Analysis of lung tumor initiation and progression using conditional expression of oncogenic K-ras. Genes Dev..

[53] Olive KP (2004). Mutant p53 gain of function in two mouse models of Li-Fraumeni syndrome. Cell.

[54] Lei J (2015). Replication-competent foamy virus vaccine vectors as novel epitope scaffolds for immunotherapy. PLoS ONE.

[55] Eisel D (2019). Cognate interaction with CD4(+) T cells instructs tumor-associated macrophages to acquire M1-like phenotype. Front. Immunol..

[56] Haberer T, Becher W, Schardt D, Kraft G (1993). Magnetic scanning system for heavy ion therapy. Nucl. Instrum. Methods Phys. Res..

[57] Puck TT, Marcus PI, Cieciura SJ (1956). Clonal growth of mammalian cells in vitro; growth characteristics of colonies from single HeLa cells with and without a feeder layer. J. Exp. Med..

[58] Franken NA, Rodermond HM, Stap J, Haveman J, van Bree C (2006). Clonogenic assay of cells in vitro. Nat. Protoc..

