# Supplementary material for: Photon versus carbon ion irradiation: immunomodulatory effects exerted on murine tumor cell lines
Source: Sci Rep. 2020 Dec 9;10:21517. doi: 10.1038/s41598-020-78577-8 (PMC7726046; doi:10.1038/s41598-020-78577-8)
Supplement: Supplementary file 1 — Supplementary Information. [file 41598_2020_78577_MOESM1_ESM.pdf]

Supplementary Material to the paper

Photon versus carbon ion irradiation – immunomodulatory effects exerted on murine tumor cell lines

Laura Hartmann[1,2,†], Philipp Schröter[1,3,4,5,†], Wolfram Osen[1], Daniel Baumann[6,7], Rienk Offringa[6,7], Mahmoud Moustafa[3,8,9,10], Rainer Will[11], Jürgen Debus[3,4,5,8,9], Stephan Brons[4,5], Stefan Rieken[3,4,5,12,‡,*] and Stefan B. Eichmüller[1,‡,*]

[1] German Cancer Research Center (DKFZ), Research Group GMP & T Cell Therapy, Heidelberg, Germany
[2] Faculty of Biosciences, Heidelberg University, Heidelberg, Germany
[3] Heidelberg University Hospital (UKHD), Department of Radiation Oncology, Heidelberg, Germany
[4] Heidelberg Institute of Radiation Oncology (HIRO), Heidelberg, Germany
[5] Heidelberg Ion-Beam Therapy Center (HIT), Heidelberg University Hospital (UKHD), Department of Radiation Oncology, Heidelberg, Germany
[6] German Cancer Research Center (DKFZ), Molecular Oncology of Gastrointestinal Tumors, Heidelberg, Germany
[7] Heidelberg University Hospital (UKHD), Department of Surgery, Heidelberg, Germany
[8] Faculty of Medicine Heidelberg (MFHD), Division of Molecular and Translational Radiation Oncology, Heidelberg, Germany
[9] German Cancer Consortium (DKTK) Core-Center Heidelberg, German Cancer Research Center (DKFZ), Heidelberg, Germany
[10] Suez Canal University, Department of Clinical Pathology, Ismailia, Egypt
[11] German Cancer Research Center (DKFZ), Genomics and Proteomics Core Facility, Heidelberg, Germany
[12] Department of Radiation Oncology, University Medical Center Göttingen, Göttingen, Germany

[†,‡] These authors contributed equally

* Correspondence and requests for material should be addressed to SBE (s.eichmueller@dkfz.de) and SR (stefan.rieken@med.uni-goettingen.de)

Supplementary Methods

Apoptosis/Necrosis assay
Twelve, 36 and 60 h after irradiation, cells were harvested and pooled with supernatants and stained for apoptotic/necrotic cells using the FITC Annexin V or PE Annexin V Apoptosis Detection Kit with 7-AAD (Biolegend) according to manufacturer's instructions. Samples were acquired using a BD FACSCanto II Flow Cytometer (BD Biosciences) run with FACS-Diva software version 6.2 (BD Biosciences) and analyzed with FlowJo V10.4.2 (Tree Star).

RNA isolation and quantitative PCR
Irradiated cells were shock-frosted in liquid nitrogen. RNA was isolated using the RNeasy Mini Kit (Qiagen, Hilden, Germany) according to manufacturer´s instructions and subjected to reverse transcription using the Transcriptor First Strand cDNA Synthesis Kit (Hoffmann-La Roche, Basel, Switzerland). For quantitative PCR, 2x Power SYBR® Green PCR Master Mix (Thermo Fisher Scientific), 10 ng cDNA, 200 nM forward and reverse primer were resolved in nuclease-free water resulting in a total volume of 20 µl. Selected genes were amplified using the ABI 7300 Real-time PCR System (Applied Biosystems, Foster City, USA). The relative fold gene expression was calculated with the $2^{-\Delta\Delta Ct}$ method normalized to the housekeeping gene Ppia. Primers used for quantitative PCR are listed in Supplementary Table S2.

Cell viability assay
Cell viability assays were performed using the Colorimetric Cell Viability Kit III (XTT) (PromoCell, Heidelberg, Germany). Briefly, in parallel to the cytotoxicity assay, irradiated EO771/Luci/OVA cells were incubated for 18 h without CTLs. Then, 50 µl reaction solution were added per well followed by an incubation period of 3 h. The optical density (OD) at 450 nm and a background wavelength of 630 nm, which was subtracted from the OD450 value, were measured using a CLARIOstar Plus Microplate Reader (BMG Labtech, Ortenberg, Germany). Finally, the mean OD of a blank control (medium) was subtracted from the OD values of the samples.

Supplementary Table S1: Monoclonal antibodies and isotype controls used for flow cytometry.

Specificity	Conjugate	Cat. No.	Manufacturer	Clone
H2-D^b	PerCP/Cy5.5	111517	Biolegend	KH95
H2-K^b	FITC/<br>PE/Cy7	116505/<br>116519	Biolegend	AF6-88.5
CD274 / PD-L1	Brilliant Violet 785/<br>PE	124331/<br>124307	Biolegend	10F.9G2
CD73 / NT5E	APC/Fire 750	127221	Biolegend	TY/11.8

Specificity	Conjugate	Cat. No.	Manufacturer	Clone
Isotype Ctrl.	PerCP/Cy5.5	400337	Biolegend	MPC-11
Isotype Ctrl.	FITC/<br>PE/Cy7	400209/<br>400253	Biolegend	MOPC-173
Isotype Ctrl.	Brilliant Violet 785/<br>PE	400647/<br>400607	Biolegend	RTK4530
Isotype Ctrl.	APC/Fire 750	400455	Biolegend	RTK2071

Supplementary Table S2: Primers used for quantitative PCR

Target	Primer	Sequence (5'-3')	Product size [bp]	Efficiency[(*)] [%]	Source
Ppia	Ppia_fwd	GAGCTGTTTGCAGACAAAGTTC	125	95.6	PrimerBank ID 6679438c1
	Ppia_rev	CCCTGGCACATGAATCCTGG			
CD274	PD-L1_fwd	TCGCCTGCAGATAGTTCCC	133	94.6	Schröter et al. 2020
	PD-L1_rev	TGACGTTGCTGCCATACTCC			
CD73	NT5E_fwd	GCATTCCTGAAGATGCGACC	91	100.7	Schröter et al. 2020
	NT5E_rev	ATCGTTCTCCCGAGTTCCTG			
MHC-I (H2-D^b)	H2-D1_fwd	GTGCTGCAGAGCATTACAAGG	110	90.6	Schröter et al. 2020
	H2-D1_rev	TGCCTTTGGGGAATCTGTGC			
MHC-I (H2-K^b)	H2-K1_fwd	TGAGAAGGAGAAACACAGGTGG	151	91.6	this paper
	H2-K1_rev	GTCACCAAGTCCACTCCAGG			

(*) Primer efficiency was determined by serial dilutions of the cDNA template. Ct values were plotted as a standard curve and the slope of this standard curve was used to calculate the primer efficiency using the following formula: Efficiency [%] = $(10^{(-1/slope)}-1)*100$

Reference:
Schröter P, Hartmann L, Osen W, Baumann D, Offringa R, Eisel D, Debus J, Eichmüller SB, and Rieken S (2020) Radiation-induced alterations in immunogenicity of a murine pancreatic ductal adenocarcinoma cell line. *Scientific Reports 10: 686.*

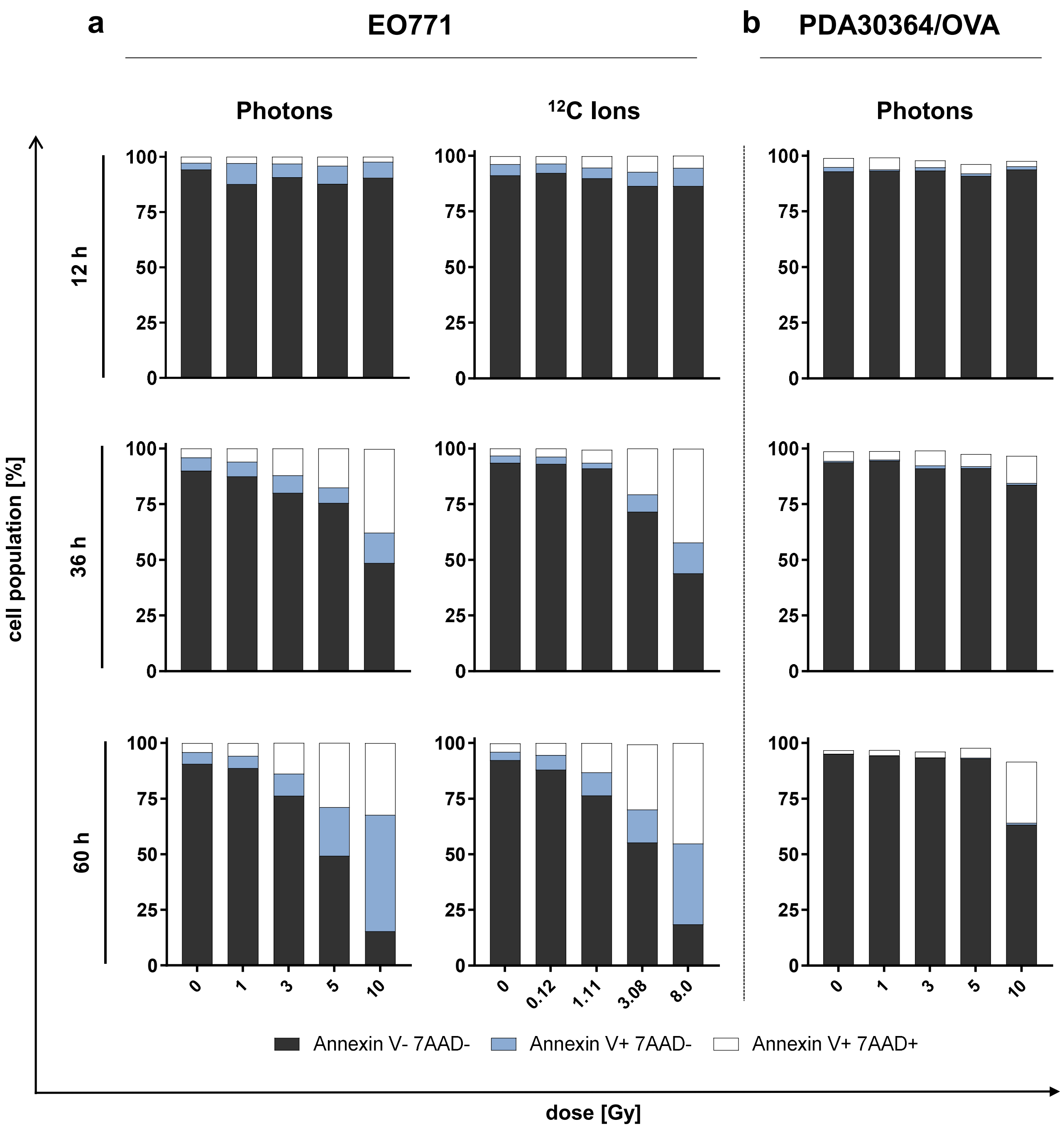

Supplementary Figure S1: Radiation-induced early and late apoptosis/necrosis on EO771 and PDA30364/OVA cells. (a) Analysis of apoptosis markers (Annexin-V/7-AAD) on EO771 cells irradiated with photons and biologically equivalent doses of carbon ions. (b) PDA30364/OVA cells were analyzed after photon irradiation as published in *Schröter et al.* (2020). Cells were classified as follows: viable cells (Annexin V- 7-AAD-), early apoptotic cells (Annexin V+ 7-AAD-), and late apoptotic/necrotic cells (Annexin V+ 7-AAD+). Representative results of one out of two independent experiments are shown for EO771. Gating strategy: cells (FSC-A vs. SSC-A) → single cells (FSC-A vs. FSC-H) → 7-AAD-A vs. Annexin V (FITC-A).

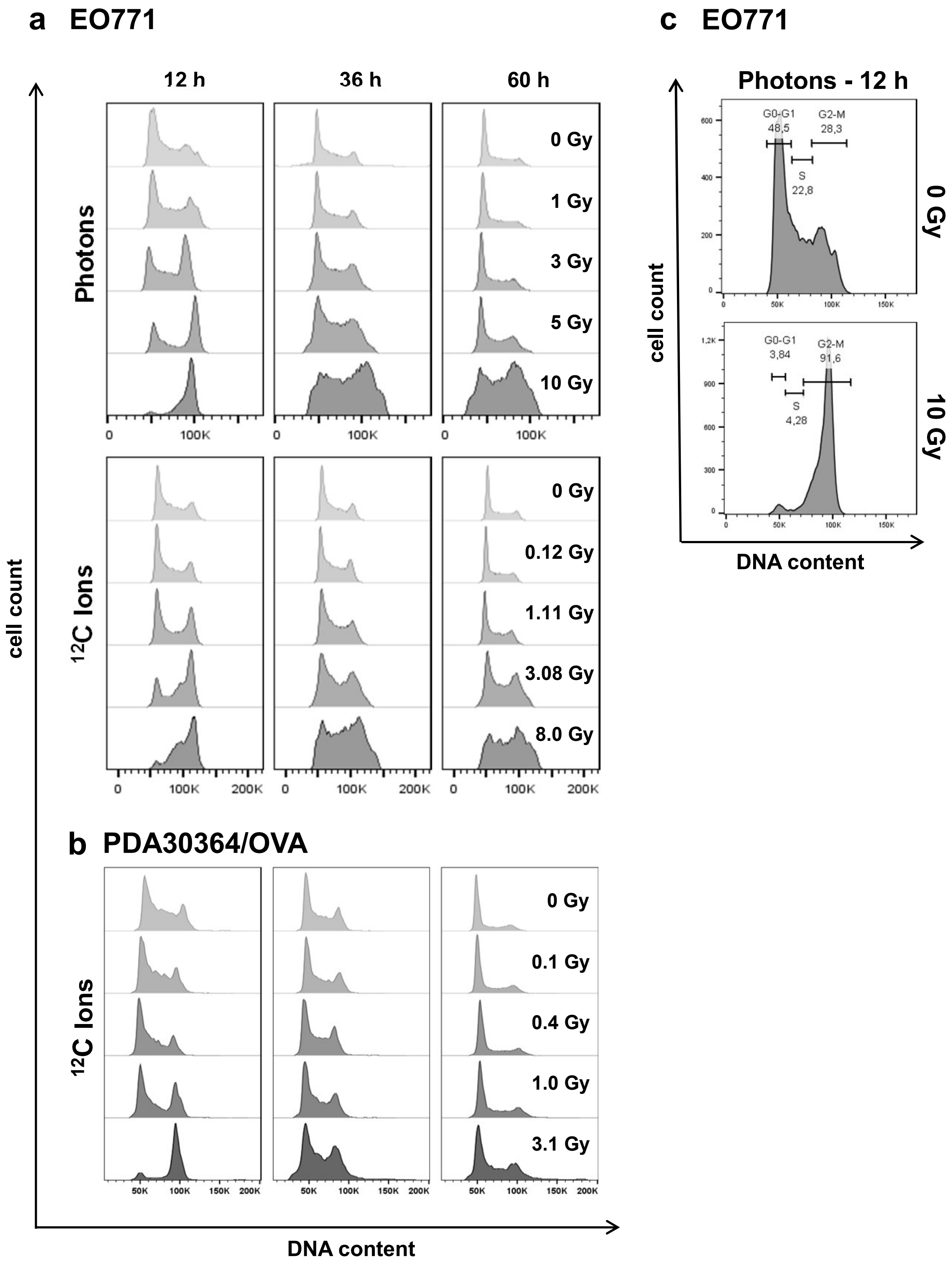

Supplementary Figure S2: Cell cycle analysis of EO771 and PDA30364/OVA cells after photon or carbon ion irradiation. Histograms of cell cycle analysis for EO771 (a) and PDA30364/OVA cells (b) 12, 36 and 60 h after irradiation with photons or carbon ions biologically equivalent to the indicated photon doses. Representative plots for definition of cell cycle phases by DNA content as measured by PI staining (c). Gating strategy: cells (FSC-A vs. SSC-A) → single cells (PI-W vs. PI-A) → PI-A vs. count.

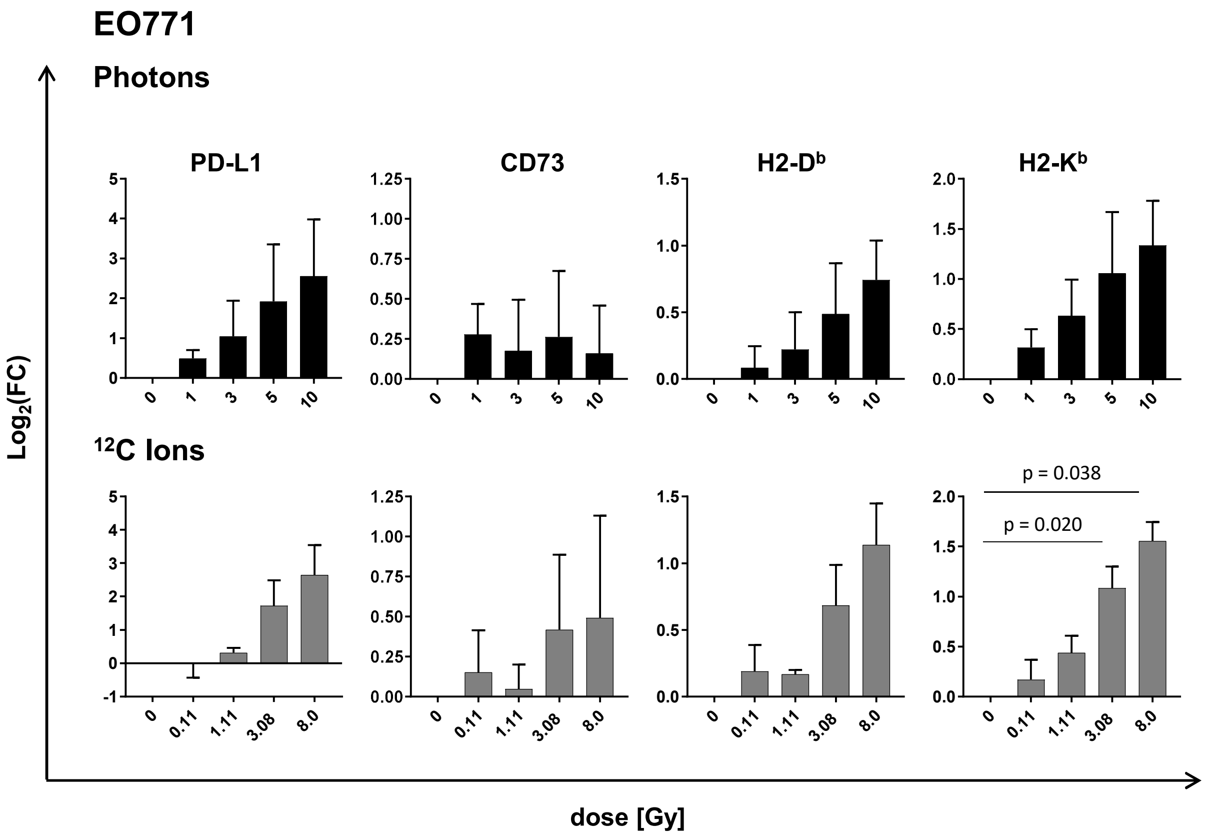

Supplementary Figure S3: Gene expression profiles of immunomodulatory surface molecules in EO771 cells after photon and carbon ion irradiation. Quantitative PCR analysis of PD-L1, CD73 and MHC-I (H2-D^b and H2-K^b) expression 36 h after irradiation with photons (top) or biologically equivalent doses of carbon ions (bottom). Target gene expression was normalized to the housekeeping gene Ppia and fold changes (FC) of target gene expression over control (0 Gy) were log_2-transformed. Mean values ± SD of three independent experiments each performed in technical triplicates are shown. The median $Log_2(FC)$ of target gene expression for each treatment was tested against the hypothetical value of 0 using a two-tailed one-sample t test and correction of P values for multiple testing was done by Holm-Bonferroni method. Multiplicity adjusted P values are shown, $\alpha = 0.05$.

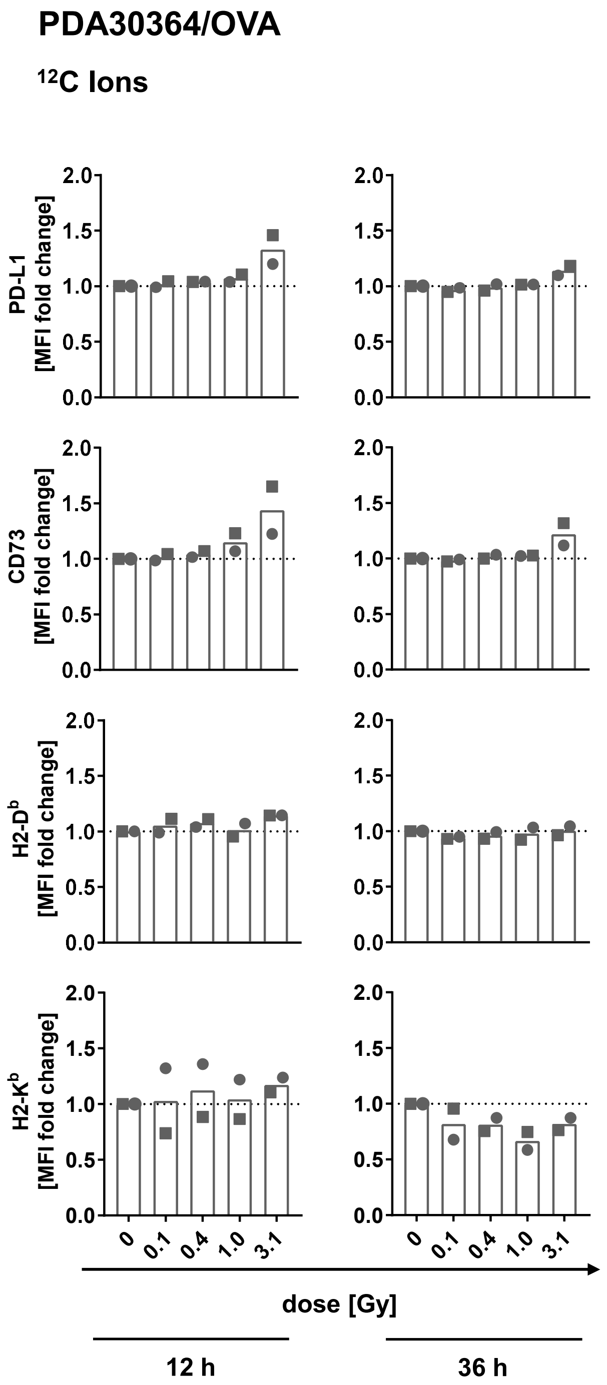

Supplementary Figure S4: Effect of carbon ion irradiation on surface expression of immunomodulatory molecules by PDA30364/OVA cells. Flow cytometric analysis of PD-L1, CD73 and MHC-I (H2-D^b / H2-K^b) cell surface expression 12 and 36 h after irradiation of PDA30364/OVA cells with carbon ion doses biologically equivalent to photon doses of 1, 3, 5, and 10 Gy. Depicted are fold changes of MFI (mean fluorescence intensity) normalized to MFI of non-irradiated cells. At least 20,000 viable cells were acquired per sample. Results of two experiments performed are shown (squares: experiment 1; dots: experiment 2).

EO771

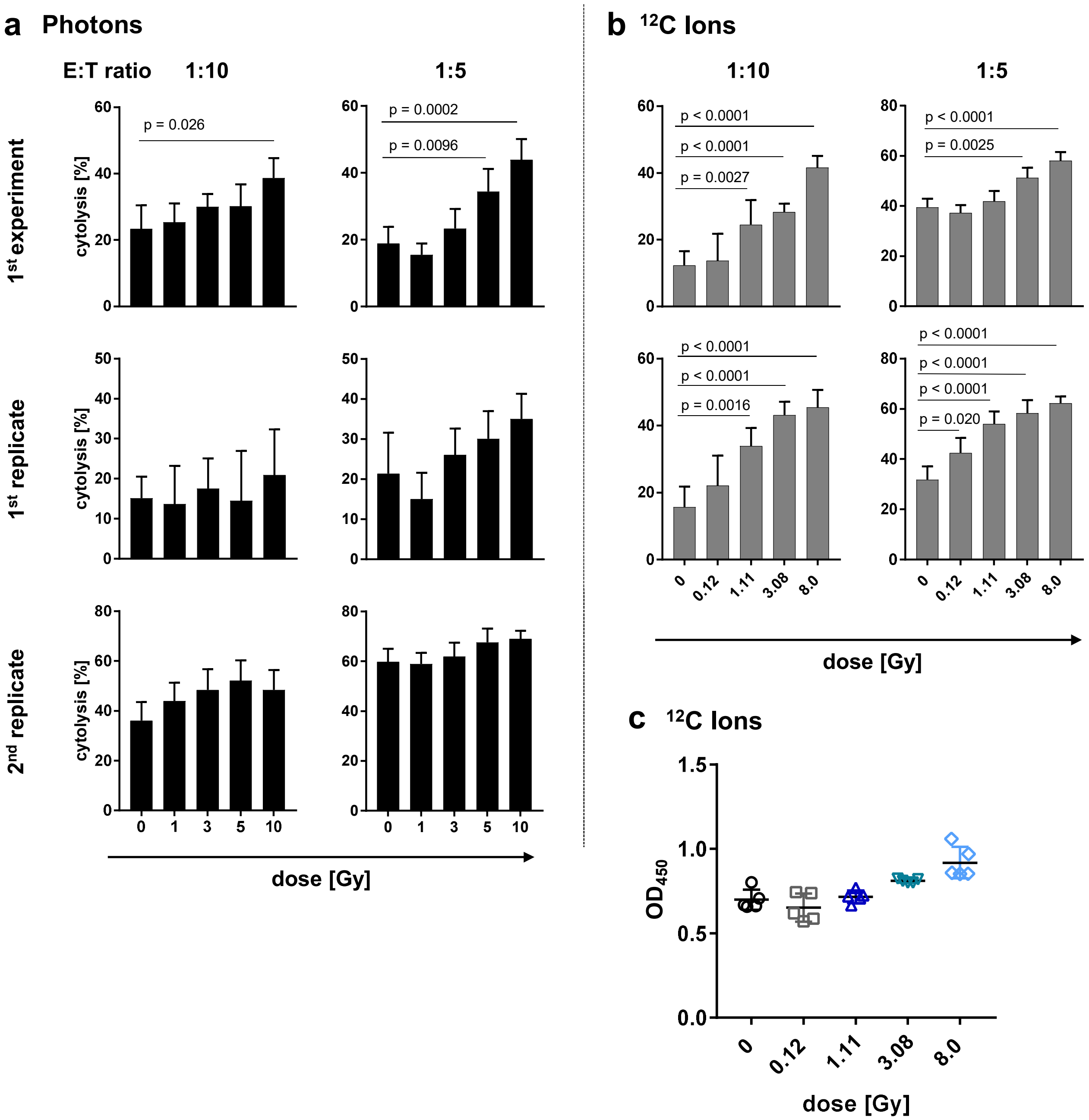

Supplementary Figure S5: Reproducibility of luciferase-based killing assay and monitoring of EO771/Luci/OVA cell viability. EO771/Luci/OVA cells were irradiated with increasing doses of photons (a) or carbon ions (b). Cytolysis of target cells by OVA-specific CTLs was measured by luciferase-based cytotoxicity assay with effector to target cell (E:T) ratios of 1:10 and 1:5 in three (photons) or two (carbon ions) independent experiments. Graphs depicted in Fig. 4a, c are included again for better comparability. (c) To control for irradiation induced cell death of target cells during luciferase-based cytotoxicity assays, cell viability of irradiated EO771/Luci/OVA, cultured for 18 h without CTLs, was measured.

PDA30364/OVA

Photons

a

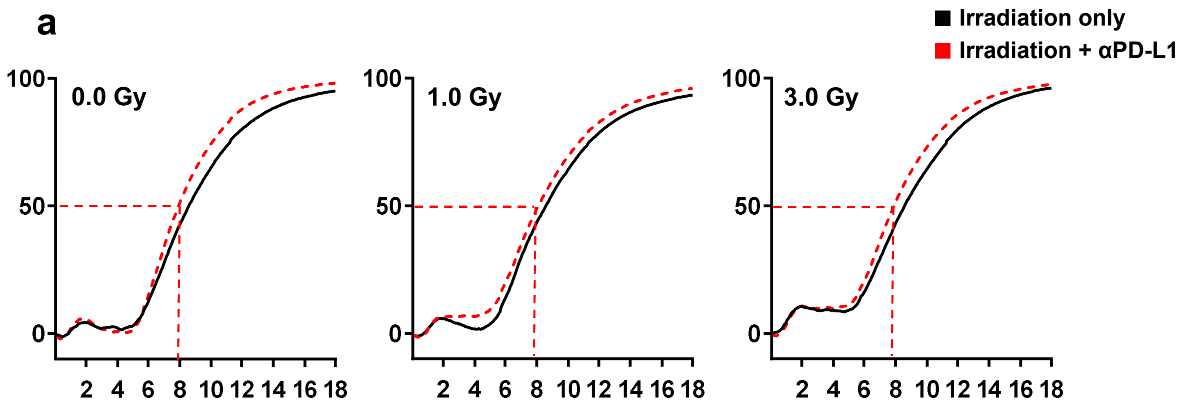

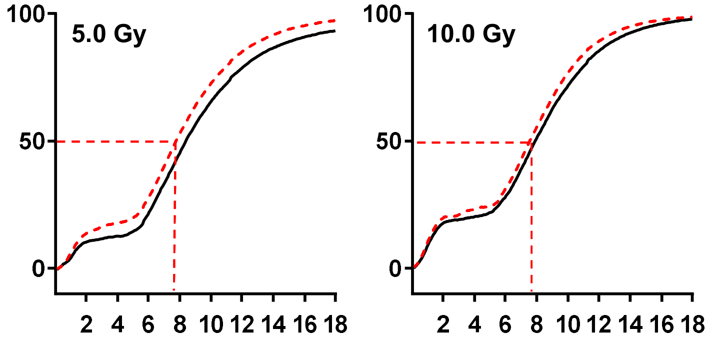

b

Photons [Gy]	KT50 [h]
	Irrad. + αPD-L1
0.0	7.93
1.0	8.09
3.0	7.93
5.0	7.76
10.0	7.59

^{12}C Ions

c

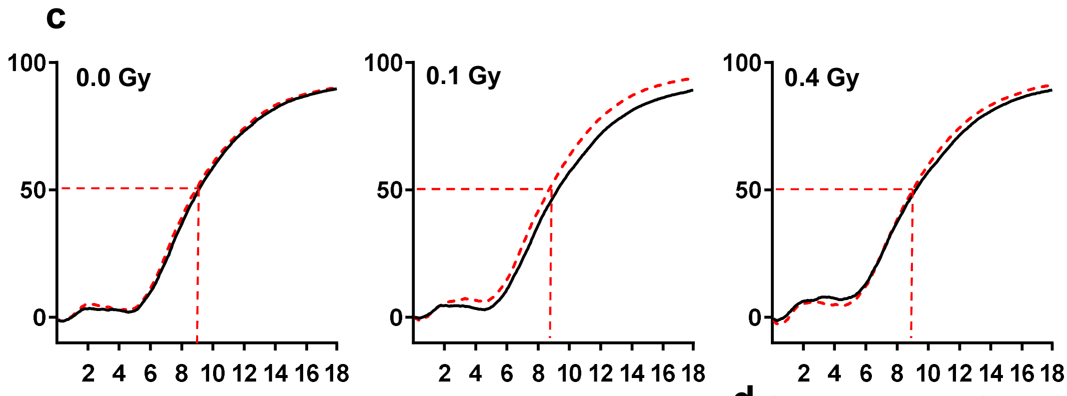

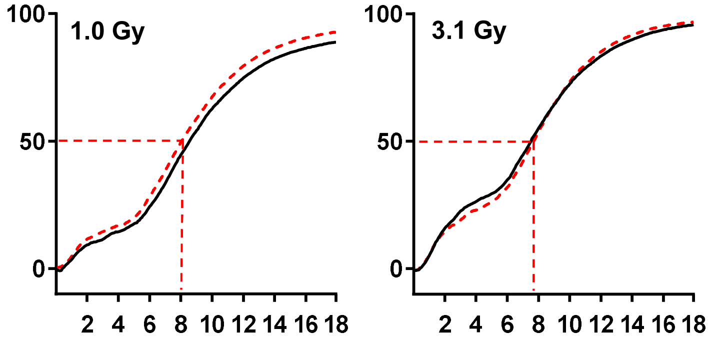

d

^{12}C Ions [Gy]	KT50 [h]
	Irrad. + αPD-L1
0.0	8.93
0.1	8.68
0.4	9.01
1.0	8.09
3.1	7.68

cytolysis [%]

T cell coculture [h]

Supplementary Figure S6. Susceptibility of PDA30364/OVA cells to OVA-specific CTL lysis co-cultured in the presence or absence of αPD-L1 mAb following photon or carbon ion irradiation. Cytolysis of PDA30364/OVA cells following irradiation with increasing photon or carbon ion doses in the presence (red dashed line) or absence (black solid line) of αPD-L1 mAb monitored for 18 h and quantified by impedance-based cytotoxicity assay (xCELLigence); effector to target cell ratio was 2.5:1; αPD-L1 was used at a concentration of 10 µg/ml (a and c). Time span elapsed until 50% of target cells underwent CTL mediated lysis was expressed as KT50 (b and d). Representative results of one out of three independent experiments performed are shown.
